# CXCR1/Akt signaling activation induced by mesenchymal stem cell-derived IL-8 promotes osteosarcoma cell anoikis resistance and pulmonary metastasis

**DOI:** 10.1038/s41419-018-0745-0

**Published:** 2018-06-18

**Authors:** Lin Du, Xiu-guo Han, Bing Tu, Min-qi Wang, Han Qiao, Shu-hong Zhang, Qi-ming Fan, Ting-ting Tang

**Affiliations:** 1 Shanghai Key Laboratory of Orthopedic Implants, Department of Orthopedic Surgery, Shanghai Ninth People’s Hospital, Shanghai Jiao Tong University School of Medicine, Shanghai, China; 2 Department of Orthopaedic Surgery, Renji Hospital, Shanghai Jiao Tong University School of Medicine, Shanghai, China; 3 Department of Orthopaedic Surgery, Shanghai Sixth People’s Hospital Affiliated to Shanghai Jiao Tong University, Shanghai, China

## Abstract

The loss of appropriate cell adhesion normally induces apoptosis via a process termed anoikis. The aim of this study was to investigate the effects of mesenchymal stem cells (MSCs) in the cancer microenvironment on the anoikis resistance and pulmonary metastasis of osteosarcoma (OS) cells, and to evaluate the critical role of the interleukin (IL)-8/C-X-C chemokine receptor (CXCR) 1/Akt-signaling pathway in these processes. Metastatic OS subtype cells, which did or did not interact with MSC-conditioned medium (MSC-CM) in vitro, were isolated from the pulmonary site and named Saos2-lung-M. Both MSC-CM and IL-8 treatment increased the anoikis resistance of Saos2 cells in vitro. Moreover, exogenous MSC-CM promoted the survival and metastasis of Saos2 cells in nude mice. Saos2-lung-M cells were more malignant and resistant to anoikis than parental cells. MSCs secreted IL-8, thereby protecting OS cells from anoikis. Blocking the IL-8/CXCR1/Akt pathway via CXCR1 knockdown inhibited the pulmonary metastasis of Saos2-lung-MSCs and prolonged the survival of tumor-bearing mice. In conclusion, MSCs enhanced OS cell resistance to anoikis and pulmonary metastasis via regulation of the IL-8/CXCR1/Akt pathway. These findings suggest that MSCs can “select for” OS cells with high metastatic potential in vivo, and highlight CXCR1 as a key target in the regulation of pulmonary metastasis of OS cells.

## Introduction

Osteosarcoma (OS) is a common primary malignancy of the bone, typically occurring in children and adolescents with an age-standardized incidence of approximately five per million cases per year^1–3^. OS originates from primitive mesenchymal bone-forming cells and often occurs in the long bones such as the proximal tibia and distal femur^4, 5^. Current OS treatment regimens consist of a combination of surgery and intensive multi-agent chemotherapy, and overall survival rates for non-metastatic OS are approximately 50–70%^6^. However, 30–40% of patients with OS experience pulmonary metastasis and relapse, which are associated with significantly poor prognoses; indeed, the overall 5-year survival rate is only about 20%^7^. Amputation of the affected limbs is often the only remaining treatment option; however, even this intervention usually fails to save patient lives due to early metastases^8^. Therefore, the development of novel techniques for preventing and treating OS metastases is highly desired.

As a barrier to metastasis, cells normally undergo apoptosis upon losing contact with their associated extracellular matrix or neighboring cells. This type of cell death is termed “anoikis”^9, 10^. Tumor cells resistant to anoikis can survive longer when unattached; therefore, these cells are involved in cell migration and tissue remodeling^11^. In addition, several studies have delineated the complex interactions between bone marrow-derived mesenchymal stem cells (MSCs) and tumor cells^12, 13^. Metaphyses are the sites of OS predilection and are rich in MSCs;^14^ these conditions facilitate the interactions between MSCs and OS cells. Previous studies, including our own work^15–17^, have indicated that MSCs could promote tumor engraftment and metastatic colonization in a rat OS model. However, the role of MSCs in the OS resistance to anoikis and the underlying associated molecular mechanisms remain unknown.

MSCs exert a proinflammatory influence by constitutively secreting cytokines into the bone marrow microenvironment^18, 19^. Interleukin (IL)-8 is one of the predominant transcriptional targets of the inflammatory signaling mediated by nuclear factor-κB, which is commonly activated in tumor microenvironments^20^. Migratory inhibitory factor-induced stromal protein kinase C β/IL-8 is essential in human acute myeloid leukemia, and introduction of targeted IL-8 small hairpin ribonucleic acid (RNA) inhibits MSC-induced acute myeloid leukemia^21^. Furthermore, Avnet et al.^22^ suggested that the OS microenvironment is a key factor in MSC activation, which promotes the secretion of paracrine factors, such as IL-8 and IL-6 that significantly influence tumor behavior. Jiang et al.^23^ showed that IL-8 promotes human OS cell invasion by regulating the phosphoinositide 3-kinase (PI3K)/protein kinase B (Akt) signaling pathway. Our previous studies also showed that expression of the IL-8-specific receptor C-X-C chemokine receptor (CXCR) 1 is upregulated in the highly metastatic OS cell line Saos2-lung^24^, and knockdown of CXCR1, which is regulated by IL-8/CXCR1/Akt signaling, increased the sensitivity of the Saos2-lung cells to cisplatin.^25^.

In the present study, we investigated the interactions between MSCs and OS cells using a living cell-tracing imaging system, and examined the influence of MSCs in the cancer microenvironment on OS cell anoikis resistance and pulmonary metastasis. Moreover, through isolation of highly metastatic OS cells, we determined an important role for the IL-8/CXCR1/Akt-signaling pathway in anoikis resistance.

## Results

### MSC-conditioned medium (CM) protected OS cells from anoikis in vitro and in vivo

**Fig. 1:**
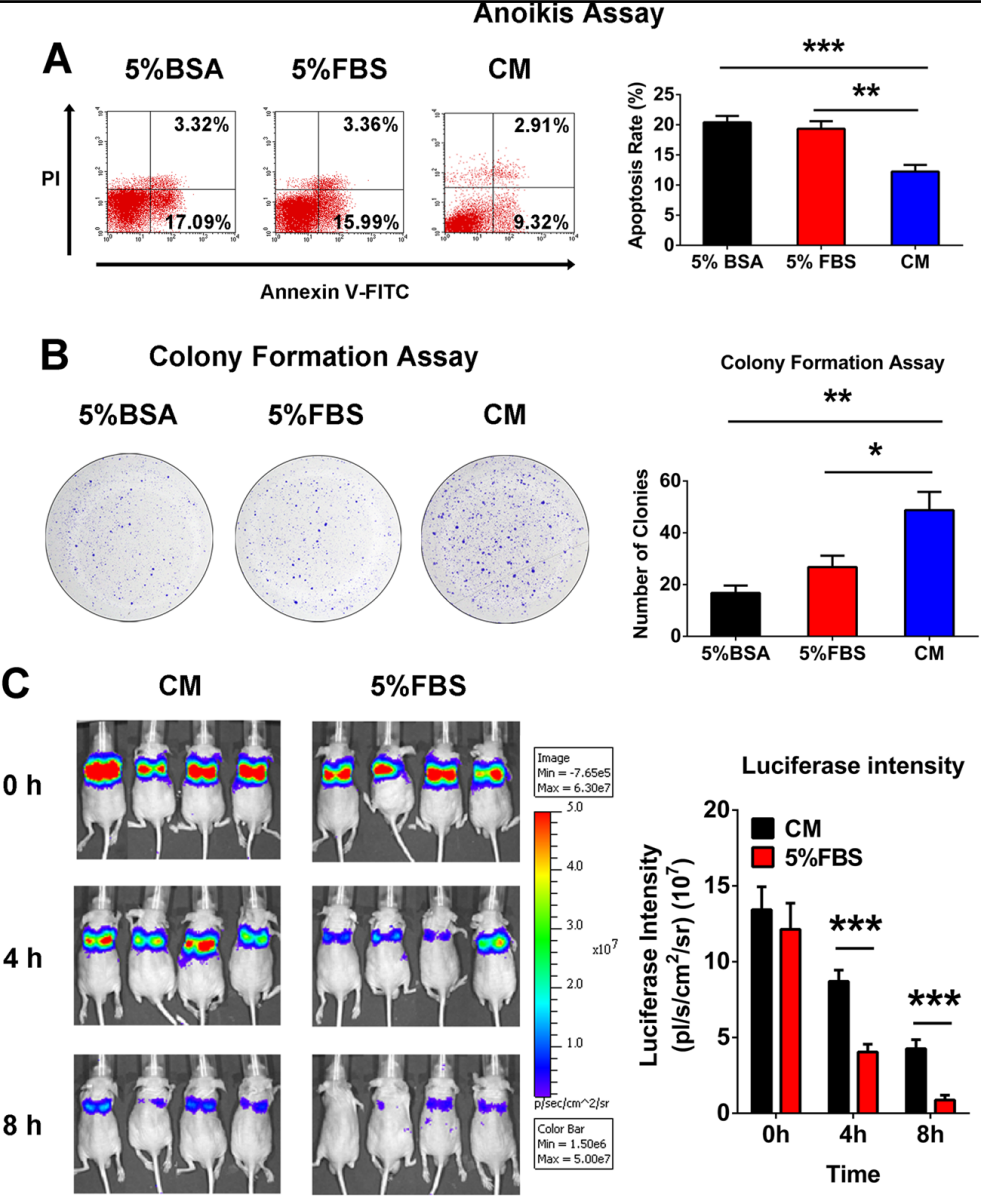
MSC-CM promotes osteosarcoma cell resistance to anoikis in vitro and in vivo. **a** Saos2 cells were cultured in suspension with 5% bovine serum albumin or DMEM containing 5% FBS or MSC-CM. The number of apoptotic cells was measured by flow cytometry. **b** Colony formation assay. **c** Monitoring of osteosarcoma cell survival in vivo using the IVIS 200 system. Data are presented as mean ± standard deviation. *p < 0.05, **p < 0.01, and ***p < 0.001. All in vitro data were obtained from at least three independent experiments. Each group contained 10 animals

To evaluate the effect of the MSC-CM on anoikis, Saos2 cells were cultured in suspension and anoikis was quantified based on the number of apoptotic cells as measured by flow cytometry. The results showed a lower rate of apoptosis in the cell population cultured in MSC-CM than in the two control groups (5% bovine serum albumin or 5% fetal bovine serum (FBS) group) (Fig. 1a). Next, 600 cells from the same groups were cultured in an adhesive state with the original stimulus to test their ability to form colonies. There were significantly more colonies detected from the MSC-CM-stimulated cells than control cells (Fig. 1b). Furthermore, we analyzed MSC-CM-treated and untreated Saos2 cells in vivo by monitoring OS cell survival in mice with an in vivo imaging system (IVIS). Similar to the in vitro results, we discovered that the MSC-CM-treated cells survived much longer than the control cells (Fig. 1c). Therefore, we concluded that MSC-CM protected the OS cells from anoikis in vitro and promoted the survival of tumor cells in vivo.

### The distribution of MSCs in OS-bearing nude mice and MSC enhancement of OS growth and metastasis

**Fig. 2:**
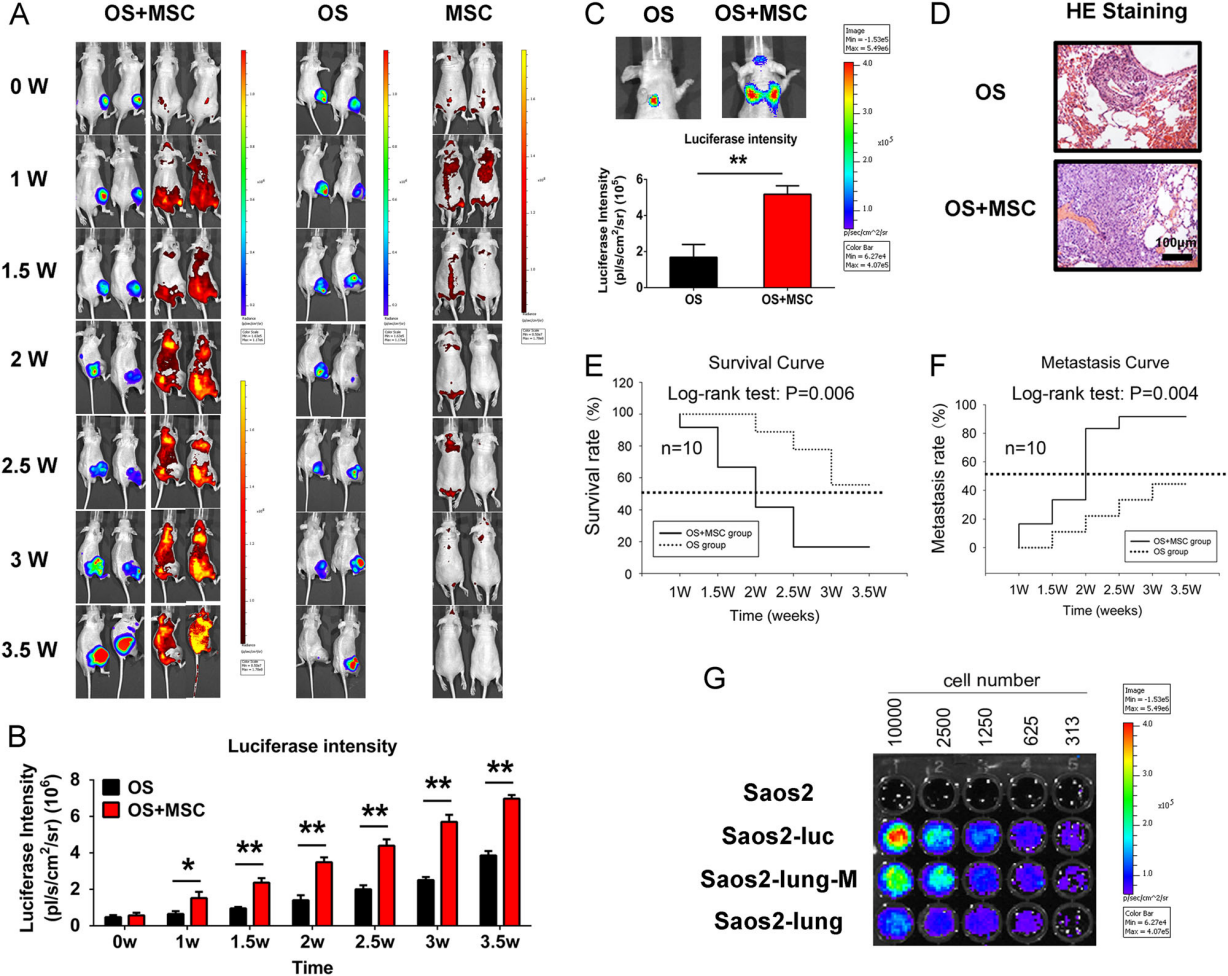
MSCs enhanced OS metastasis in OS-bearing nude mice. **a** The interactions between MSCs and OS cells were monitored in vivo using the IVIS 200 system. **b** Quantification of luciferase intensity. Data are presented as mean ± standard deviation (S.D.). *p < 0.05 and **p < 0.01. Each group contained 10 animals. **c** Pulmonary metastasis of OS was facilitated by interactions with MSCs. **d** HE staining of pulmonary metastasis of OS. **e** Survival–time curve indicating that MSCs shortened the survival of OS-bearing mice. **f** Metastasis–time curve indicating that MSCs promoted the pulmonary metastasis of OS. **g** Identification of isolated luciferase-labeled OS cells that underwent pulmonary metastasis in the presence or absence of the MSC-CM stimulus. Data are presented as mean ± S.D. *p < 0.05, **p < 0.01. All in vitro data were obtained from at least three independent experiments. Each group contained 10 animals

We established a primary OS model to verify the effect of MSCs on the growth and metastasis of Saos2 cells. After tumor formation for 4 weeks, red fluorescent protein (RFP)-labeled MSCs were injected via the tail vein into the OS model and control mice. An IVIS was used to monitor the growth of MSCs and OS cells for 3.5 weeks at half-week intervals. Figure 2a indicates that the MSCs targeted the OS with enhanced systemic distributions in OS-bearing nude mice. Quantitative data revealed that the MSCs notably promoted OS growth (Fig. 2b). Furthermore, the MSCs significantly enhanced OS metastasis (Fig. 2c). Hematoxylin and eosin (HE) staining confirmed that OS metastasis was severe in MSC-CM-treated cells (Fig. 2d). Survival and metastasis curves indicated that the MSCs facilitated pulmonary metastasis in OS-bearing nude mice and shortened their survival period (Fig. 2e, f). Next, luciferase-labeled Saos2-lung-M cells were isolated from the sites of pulmonary metastasis and identified in vitro, using Saos2-lung^25^ and Saos2-luc^26^ cells as positive controls and unlabeled Saos2 cells as the negative control (Fig. 2g). Thus, we monitored the MSC interactions with OS cells in real-time in vivo in a non-invasive manner and isolated Saos2-lung-M cells, which represent a subtype of metastatic OS cells after MSC treatment.

### Saos2-lung-M cells with IL-8/CXCR1 activation exhibited higher anoikis resistance

**Fig. 3:**
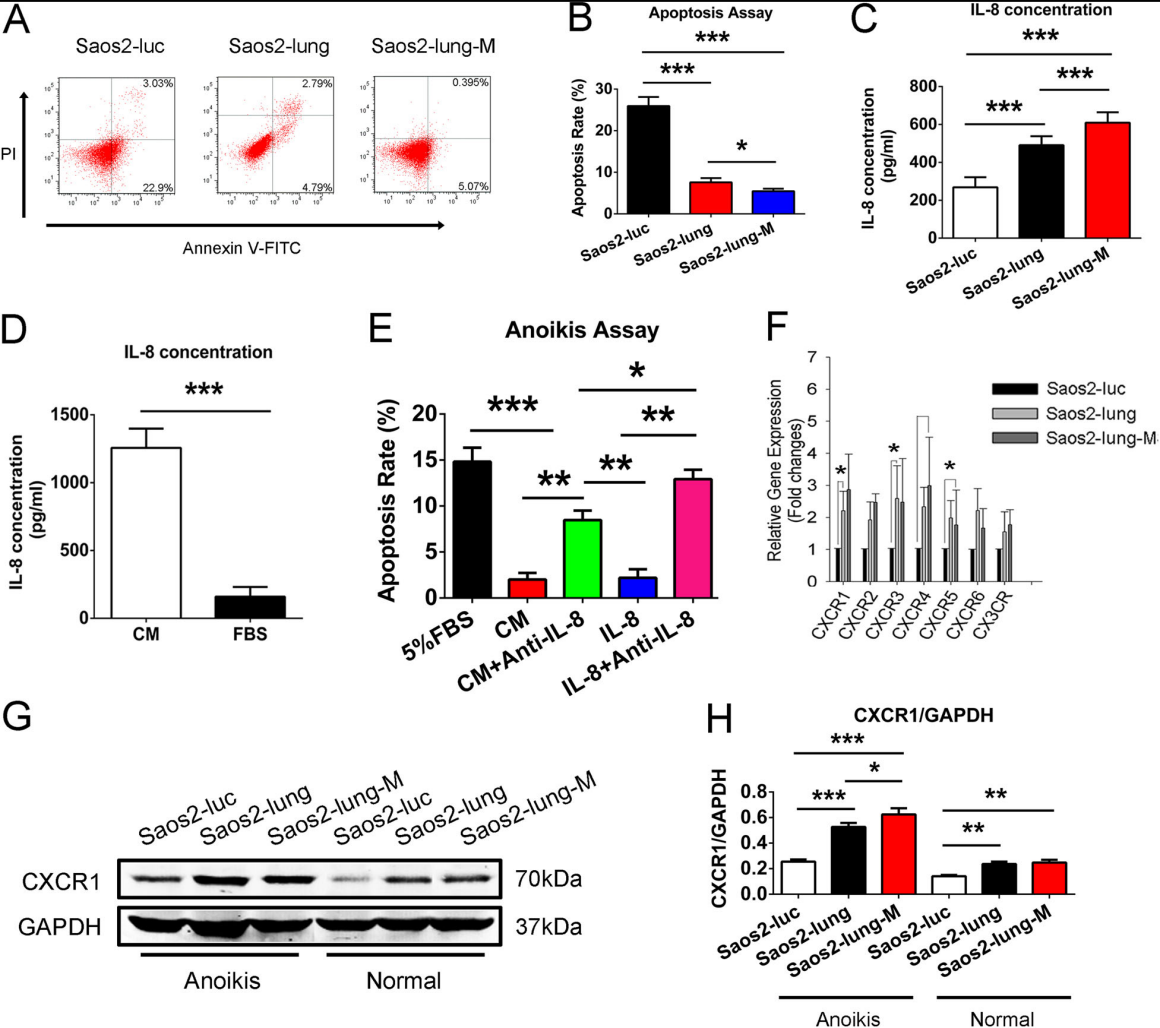
(There is a mistake in Fig.3C, the first group is Saos2-luc,not Sao2-luc,we have uploaded the revised figure 3 in the attachment of this system.)MSC-CM-derived IL-8-induced activation of CXCR1 promotes anoikis resistance in Saos2-lung-M cells. **a**, **b** Anoikis rates of Saos2-luc, Saos2-lung, and Saos2-lung-M cells cultured in suspension. **c** Comparison of IL-8 secretion by metastatic OS subtypes and parental cells. **d** Quantification of IL-8 in the MSC-CM. **e** Anoikis rate of Saos2 cells stimulated with MSC-CM, IL-8, and IL-8 or MSC-CM plus IL-8-neutralizing antibody. **f** Expression of chemokine receptors on OS cells. **g** Western blot showing CXCR1 expression in OS cells in suspension and in a normal state. **h** Quantification of western blot bands. Data are presented as mean ± standard deviation. *p < 0.05, **p < 0.01, and ***p < 0.001. All data were obtained from at least three independent experiments

When the number of cells undergoing anoikis was compared among Saos2-luc, Saos2-lung, and Saos2-lung-M cells, we found that fewer Saos2-lung-M cells underwent anoikis than Saos2-luc and Saos2-lung cells (Fig. 3a, b). Furthermore, enzyme-linked immunosorbent assay (ELISA) results show that Saos2-lung-M cells secreted more IL-8 than Saos2-luc and Saos2-lung cells (Fig. 3c), and that the MSC-CM contained more IL-8 than FBS (Fig. 3d). Further analysis revealed that both the MSC-CM and IL-8 protected OS cells from anoikis, and that IL-8-neutralizing antibody blocked this effect (Fig. 3e). Moreover, the results of polymerase chain reaction (PCR) indicated that CXCR1, an IL-8-specific receptor, was most highly expressed in Saos2-lung-M cells (Fig. 3f). Western blot analysis confirmed that the Saos-lung and Saos2-lung-M cells expressed more CXCR1 than Saos-luc cells at the protein level, both when in suspension and in a normal culture state (Fig. 3g, h). In conclusion, Saos2-lung-M cells showed higher CXCR1 activation./CXCR1 activation.

### Akt signaling acts downstream of the IL-8/CXCR1 axis in OS cell anoikis

**Fig. 4:**
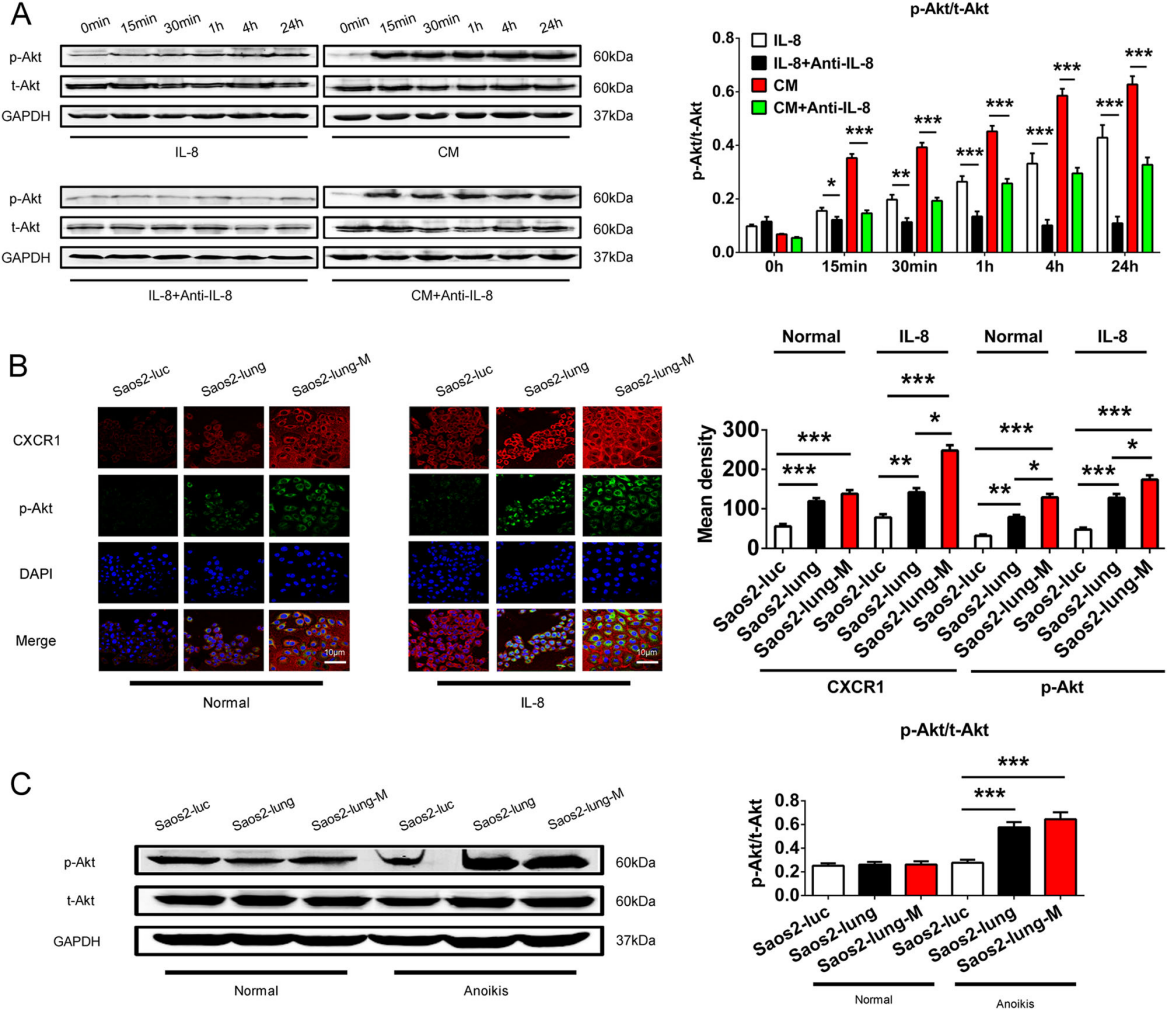
Activation of Akt in Saos2 cells from IL-8 and MSC-CM stimulus. **a** Phosphorylation of Akt protein in Saos2 cells following IL-8 or MSC-CM stimulation with or without IL-8-neutralizing antibody. **b** Immunofluorescence staining of CXCR1 and p-Akt in Saos2-luc, Saos2-lung, and Saos2-lung-M cells stimulated with or without IL-8. **c** Western blot of phosphorylated-Akt in Saos2-luc, Saos2-lung, and Saos2-lung-M cells in suspension and in a normal state. All data were obtained from at least three independent experiments. Data are presented as mean ± standard deviation. *p < 0.05, **p < 0.01, and ***p < 0.001. All data were obtained from at least three independent experiments

The MSC-CM- and IL-8-stimulated phosphorylation of Akt protein was induced in suspended Saos2 cells; however, IL-8-neutralizing antibody could block the phosphorylation of Akt (Fig. 4a). Immunofluorescence staining of IL-8-stimulated Saos2-luc, Saos2-lung, and Saos2-lung-M cells revealed that CXCR1 and p-Akt expression was more strongly upregulated in Saos2-lung-M cells than in Saos2-luc and Saos2-lung cells (Fig. 4b). Analysis of p-Akt protein levels in cells in suspension yielded parallel results (Fig. 4c). Therefore, the Akt-signaling pathway was activated in suspended OS cells by IL-8 in an anoikis-inducing environment.

### CXCR1 knockdown inhibited OS growth and metastasis in vivo

**Fig. 5:**
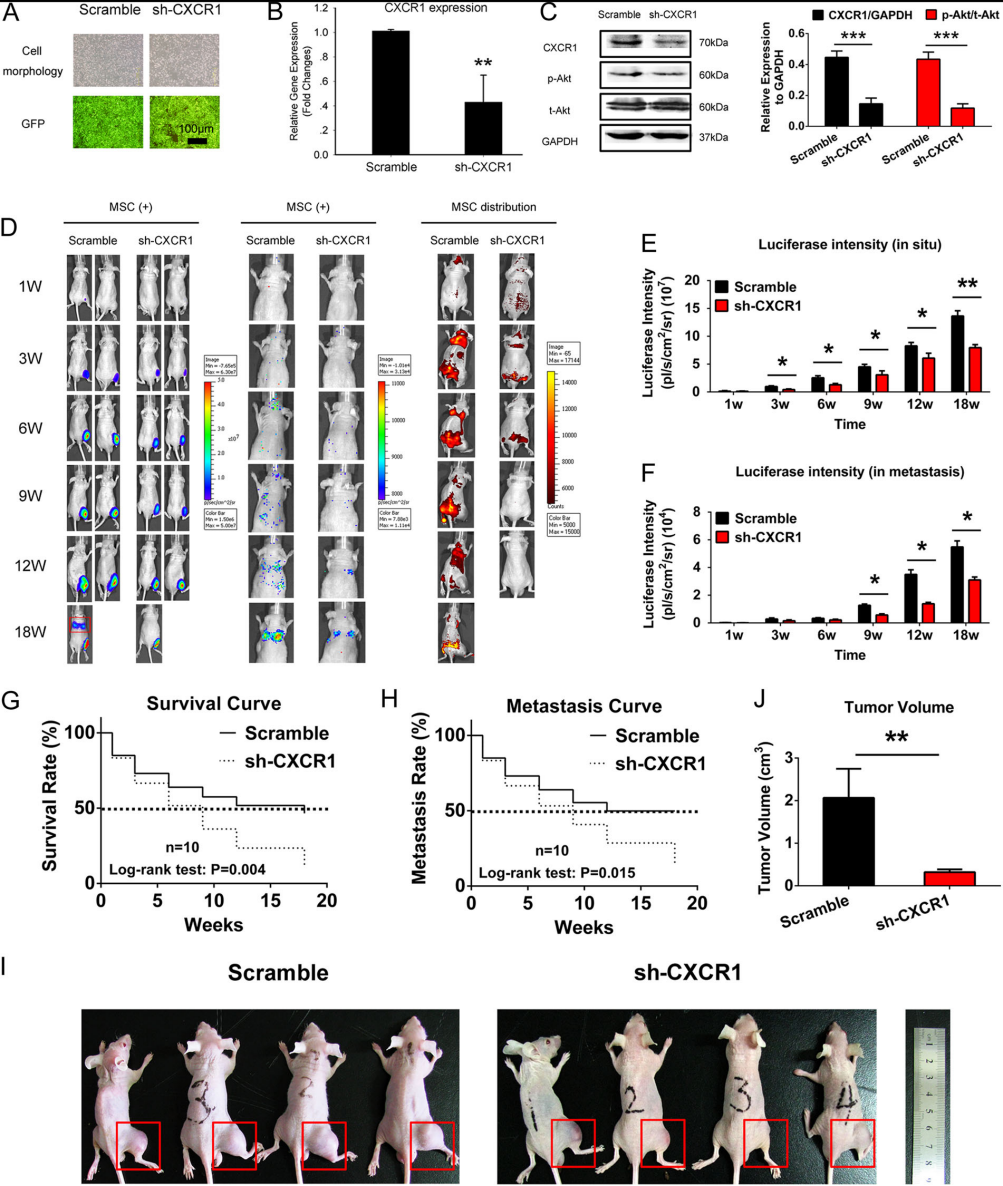
Metastasis of OS was inhibited by CXCR1 knockdown in Saos2-lung-M cells in vivo. **a** Cultured Saos2-lung-M cells with CXCR1 interference; GFP was used to label positive cells. **b** Confirmation of CXCR1 knockdown at the mRNA level. **c** Confirmation of CXCR1 knockdown at the protein level, and the phosphorylation of Akt. **d** Real-time monitoring of CXCR1 knockdown and scrambled control Saos2-lung-M cells or RFP-labeled MSCs in vivo using the IVIS 200 system. **e** Quantification of luciferase intensity in tumors in situ. **f** Quantification of luciferase intensity in metastasized tumors. **g** Survival of tumor-bearing mice containing CXCR1-knockdown and scrambled control Saos2-lung-M cells. **h** Comparison of lung metastasis between CXCR1 knockdown and scrambled control Saos2-lung-M cells. **i**, **j** Tumor volume was measured and calculated at week 18 in CXCR1-knockdown and scrambled control Saos2-lung-MSC-bearing mice. Data are presented as mean ± S.D. *p < 0.05, **p < 0.01, and ***p < 0.001. All in vitro data were obtained from at least three independent experiments. Each group contained 10 animals

Knockdown of CXCR1 expression was performed in Saos2-lung-M cells (Fig. 5a), and the successful downregulation of CXCR1 expression was confirmed by real-time PCR (Fig. 5b). CXCR1 protein expression, as well as p-Akt protein expression, also decreased (Fig. 5c). We then established a primary OS model and introduced CXCR1 knockdown or scrambled control Saos2-lung-M cells with RFP-labeled MSCs by intramedullary tibia injection. Optical imaging in vivo revealed that CXCR1 knockdown restrained OS growth and metastasis in mice, and the MSCs in the CXCR1-knockdown Saos2-lung-M-bearing nude mice completely disappeared at week 9, while the scrambled control cells were maintained for 18 weeks (Fig. 5d–f). Survival curves further indicated that CXCR1 knockdown prolonged the survival of OS-bearing nude mice (Fig. 5g), and pulmonary metastasis curves obtained using optical imaging indicated that CXCR1 knockdown suppressed lung metastasis (Fig. 5h). Accordingly, the tumor volume was smaller in the CXCR1 knockdown group than in the control (Fig. 5i, j). Therefore, we concluded that CXCR1 knockdown inhibited OS cell growth and metastasis and blocked the interactions between MSCs and OS cells in vivo.

### CXCR1 knockdown shortened the survival of OS cells in vivo and resulted in downregulation of CXCR1 and p-Akt expression in tumor samples

**Fig. 6:**
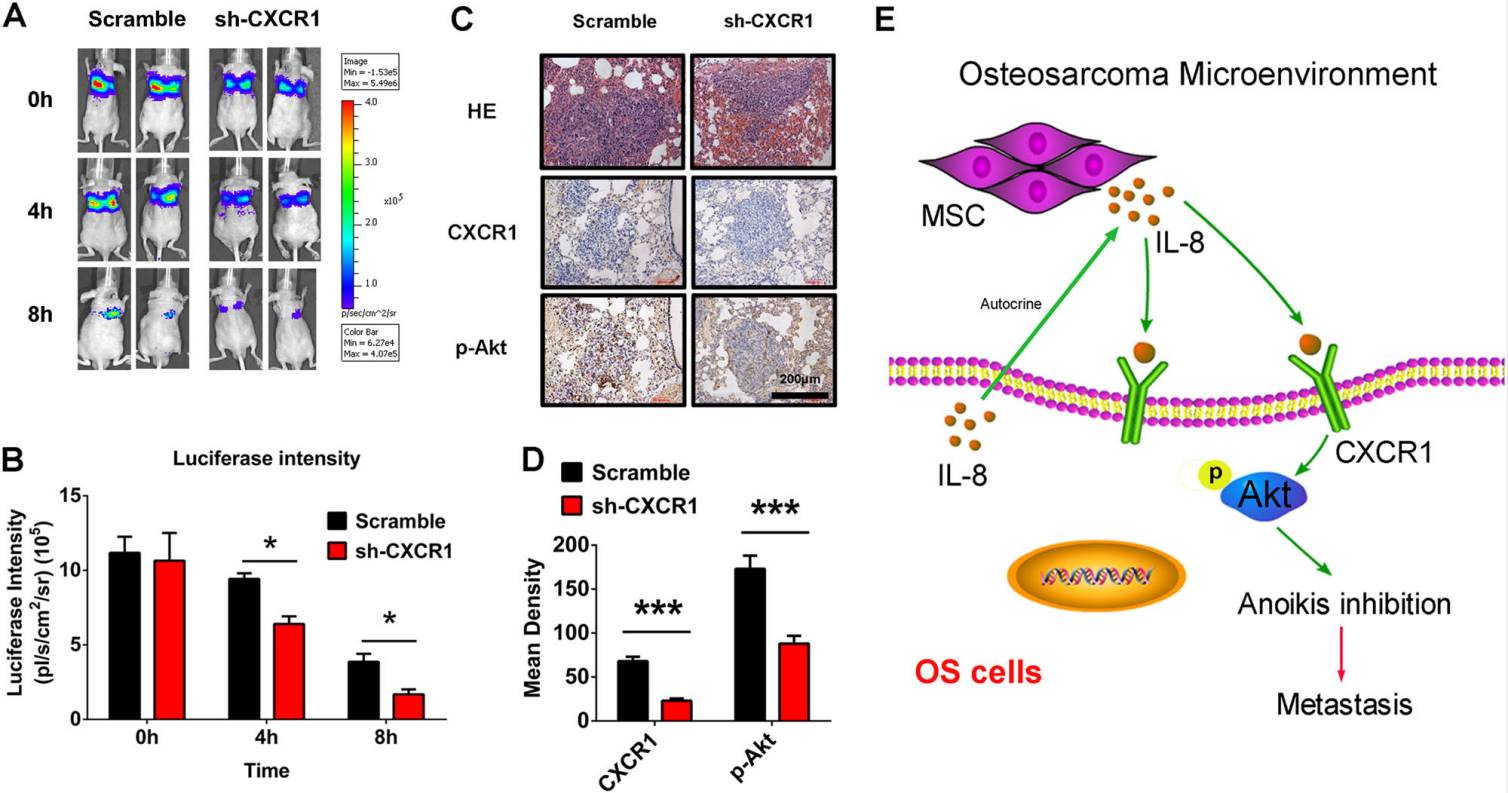
Effect of CXCR1 knockdown on OS cell survival in vivo and immunohistochemistry of CXCR1 and p-Akt. **a** Survival of Saos2-lung-M cells in vivo at 0, 4, and 8 h after CXCR1 knockdown. **b** Quantification of luciferase intensity. **c** Tissue sections were prepared from OS pulmonary metastases from nude mice at week 4. Hematoxylin and eosin staining of tumors, and immunohistochemical staining for CXCR1 and p-Akt expression. **d** Quantification of positive staining. Results are expressed as the mean density of positive staining. **e** Schematic of the proposed mechanisms of enhanced OS resistance to anoikis via IL-8/CXCR1/Akt signaling. According to the results of this study, MSC-secreted IL-8 and autocrine IL-8 of OS itself upregulate p-Akt expression through CXCR1 activation, resulting in enhanced OS cell resistance to anoikis and the promotion of lung metastasis. Data are presented as mean ± standard deviation *p < 0.05 and ***p < 0.001. Each group contained 10 animals

We analyzed the survival of CXCR1-knockdown and scrambled control Saos2-lung-M cells in vivo and found that the CXCR1-knockdown cells survived for shorter periods than the control cells (Fig. 6a, b). Metastasis tumor tissues collected as of 4 weeks post-injection of CXCR1-knockdown and scrambled control Saos2-lung-M cells were immunostained for CXCR1 and p-Akt. HE staining revealed smaller metastatic nodes, and immunostaining showed reduced expression of CXCR1 and p-Akt in the CXCR1-knockdown mice compared to the control mice (Fig. 6c, d). These data suggest that CXCR1 knockdown significantly reduced the tumor size and metastasis rate, as well as decreased the expression of CXCR1/Akt (Fig. 6e).

## Discussion

OS is a primary malignant tumor, and has the highest incidence of all tumors treated in the department of orthopedics, characterized by a high rate of metastasis^27^. Anoikis is regarded as one of the mechanisms regulating metastasis, and is thus expected to be closely related to OS metastasis as well. Besides the OS cells themselves, the OS tumor microenvironment may have a strong relationship with anoikis regulation. We have observed that the predilection site of OS is also rich in bone marrow MSCs, and a previous study revealed that complex interactions occur between MSCs and tumor cells^28^. Although MSCs are known to play an important role in promoting tumor metastasis^29^, it is still unresolved as to whether MSCs can promote tumor cell resistance to anoikis and the colonization of distant tissues. Based on the specific characteristic of OS, we believe that OS is a good disease model with which to explore whether bone marrow MSCs can facilitate tumor cell metastasis by regulating anoikis.

In the present study, we confirmed that the active components in the MSC-CM inhibited OS cell anoikis, including IL-8. By analyzing the chemokine receptor family genes in metastatic OS cell subtypes obtained during our study, we found that the chemokine receptor CXCR1 is more highly expressed in Saos2-lung-M and Saos2-lung cells than in parental OS cells. However, many chemokine receptor ligands are not unique to the chemokine receptor family;^30^ that is, these chemokine receptors are not highly specific and can be activated by more than one component. CXCR1 has only been shown to have two ligands, IL-8 and C-X-C motif chemokine ligand (CXCL)6, with high affinity only for IL-8 interactions^31^.

It is crucial to further elaborate the mechanisms driving this phenomenon. As a key protein in the regulation of cell survival, Akt protein plays an important role in anoikis^32^. Phosphorylation of Akt protein can produce a series of biological effects^33^. Previous studies have demonstrated a relationship between IL-8 and Akt protein and uncovered the IL-8/CXCR1/Akt pathway^34^. Based on this background, we focused on the potential effect of IL-8 on OS cell anoikis, revealing that IL-8 could inhibit OS cell anoikis by activating Akt. Furthermore, MSC-CM treated with IL-8-neutralizing antibody also inhibited OS cell anoikis. However, the influence of IL-8-neutralizing antibody treatment of MSC-CM was stronger than that in a medium containing IL-8. Therefore, there may be active components secreted by MSCs that help OS cells escape from anoikis in addition to IL-8. Our previous study showed that MSCs in the bone microenvironment promoted OS progression and protected tumor cells from drug-induced apoptosis through IL-6/STAT3 signaling^35^.

Although the malignancy of both Saos2-lung-MSC and Saos2-lung cells was significantly higher than that detected in the Saos2-luc parental cells, the metastatic potential of Saos2-lung-M cells was significantly higher than that of Saos2-lung cells. This suggests that MSCs play a role as “selectors” in the process of OS metastasis, perhaps by secreting IL-8 to upregulate the expression of the CXCR1/Akt signal pathway components in OS cells (Fig. 6e). Another potential mechanism involves autocrine effects, which have been shown to be important in the self-regulation of tumor cells^36^. We also found that IL-8 production was highly upregulated in both metastatic Saos2-lung-M and Saos2-lung cells.

In summary, the present study showed that MSCs enhanced OS cell resistance to anoikis, survival, and metastasis in an IL-8/CXCR1/Akt pathway-regulated manner. By blocking IL-8/CXCR1/Akt signaling via CXCR1 knockdown in tumor cells, the pulmonary metastasis of OS was inhibited and the survival of OS-bearing nude mice was prolonged. These results indicate that CXCR1 is a key target in the regulation of the pulmonary metastasis of OS cells.

## Materials and methods

### Main materials

A human IL-8 ELISA kit was purchased from R&D systems (Minneapolis, MN, USA, S8000C). Antibodies against p-Akt (4060S), t-Akt (4685S), and GAPDH (2118S), and secondary antibodies (5151S) were purchased from Cell Signaling Technology, Inc. (CST; Danvers, MA, USA). Antibodies against CXCR1 were purchased from Sigma (Darmstadt, Germany, SAB2700216). IL-8 was purchased from PeproTech., Inc. (Rocky Hill, NJ, USA).

### OS cell culture

Saos2 cells were purchased from the Chinese Academy of Sciences (Shanghai, China). This cell line was identified by the Genetic Testing Biotechnology Corporation (Suzhou, China) using short tandem repeat markers. Saos2-luc cells are luciferase-labeled Saos2 cells (Saos2-luc), which were described in our previous report^26^. The Saos2-lung cell line, which is derived from the Saos2-luc line, is a highly metastatic and chemoresistant human OS cell line^24, 25^. All cells were grown in Dulbecco’s modified Eagle’s medium (DMEM; Hyclone, Tauranga, New Zealand) supplemented with 10% FBS (Hyclone), 100 U/mL penicillin, and 100 μg/mL streptomycin in a 5% CO_2_ humidified atmosphere at 37 °C.

### MSC culture and preparation of MSC-CM

MSCs were obtained from proximal femurs during orthopedic surgery as previously described^35^ in accordance with the ethical guidelines of the Shanghai Ninth People’s Hospital, Shanghai, China. To prepare the MSC-CM, MSCs were grown to 80% confluence in 10-cm dishes in α-minimal essential medium in a 5% CO_2_ humidified atmosphere at 37 °C. The medium was discarded and the cells were further cultured in DMEM containing 5% FBS for 24 h. The medium was then collected, centrifuged at 1000 × *g* for 10 min, and passed through 0.22-μm filters (Millipore, Billerica, MA, USA). All cells were used within five passages.

In addition, we transfected a lentiviral vector system carrying the RFP gene into MSCs to facilitate imaging of MSCs in vivo.

### Isolation of highly metastatic Saos2-lung-M cells

Four-week-old male BALB/c nude mice were injected with 1 × 10^7^ Saos2-luc cells in the right proximal tibia and with 1 × 10^7^ RFP-labeled MSCs via the tail vein. Nude mice with pulmonary metastasis detectable by an IVIS (PerkinElmer, Waltham, MA, USA) were sacrificed when moribund. The lungs were then perfused with phosphate-buffered saline (PBS), excised, finely minced, and incubated for 1 h in DMEM with 150 IU/mL collagenase type IV (Sigma-Aldrich, St. Louis, MO, USA). Single-cell suspensions were prepared by repeatedly aspirating the mixture through a 10-mL syringe and then filtering through a nylon mesh. The cells were then pelleted at 1000 rpm, washed in PBS, and plated on 6-cm tissue-culture plates in complete medium with 200 μg/mL G418 (ref. ^26^) to select for OS cells. The OS cells were allowed to outgrow and form colonies for a few weeks and were then passaged 40 times. The resulting passaged OS cells were named Saos2-lung-M cells.

### Analysis of anoikis in vitro

OS cells were grown to confluence in 10-cm tissue-culture dishes unless otherwise indicated. Trypsinized cells (3 × 10^6^) were quantified and plated on 10-cm dishes coated with poly-hydroxyethylmethacrylate (HEMA), which facilitated maintenance of cells in a suspended state^37^. Poly-HEMA plates were made by adding 4 mL of a 10 mg/mL solution of poly-HEMA (Aldrich Chemical Co., Milwaukee, WI, USA) in ethanol to dishes and then drying the dishes in a tissue-culture hood. This coating and drying was repeated, followed by extensive PBS washes. After 24 h, the cells were collected from the poly-HEMA dishes. Anoikis was measured by flow cytometry using Annexin V/propidium iodide double-immunofluorescent staining. Apoptotic events were analyzed as previously described^25, 38^.

### Analysis of OS cell survival in vivo

Four-week-old male BALB/c nude mice were injected via the tail vein with 1 × 10^7^ Saos2-luc cells treated with 5% FBS or MSC-CM. Surviving OS cells in vivo were detected by bioluminescence at 0, 4, and 8 h. In addition, nude mice were injected via the tail vein with CXCR1-knockdown or scrambled Saos2-lung-M cells. OS cell survival in vivo was also detected by bioluminescence at 0, 4, and 8 h.

### Colony formation assay

Three six-well plates were seeded with Saos2 cells at a concentration of 600 cells per well in 2 mL media. After overnight incubation, each well was treated with 5% bovine serum albumin, 5% FBS, or MSC-CM. After a 14-day incubation, the media were aspirated from each well and the cells were stained with 1% crystal violet for 30 min. The number of colonies in each well was counted with plates tested in duplicate as described in a previous study^39^.

### ELISA

Saos2, Saos2-lung, and Saos2-lung-M cells were plated in six-well plates at 1 × 10^4^ cells per well. The next day, the medium was replaced with 2 mL fresh serum-free DMEM per well, and the culture supernatants were collected after 24 h. IL-8 concentrations in the culture supernatants, as well as in the MSC-CM and normal medium, were measured by ELISA according to the manufacturer’s protocols (R&D Systems, Minneapolis, MN, USA).

### RNA isolation and real-time PCR

Total RNA was isolated from Saos2, Saos2-lung, and Saos2-lung-M cells using an RNeasy Mini Kit (Qiagen, Dusseldorf, Germany), and complementary DNA was synthesized using the iScript cDNA Synthesis Kit (Bio-Rad, Hercules, CA, USA). Subsequently, real-time PCR was performed using an ABI 7500 Sequence Detection System (Thermo Scientific, Waltham, MA, USA) and SYBR Premix Ex Taq (Takara, Dalian, Liaoning, China). All procedures were performed according to the manufacturer’s protocols and a previous study^40^. Primer sequences are listed in Table 1.

**Table 1:** Sequences of primers used in real-time PCR

Gene	Primer sequences (5′-3′)	
CXCR1	Forward	CTGAGCCCCAAGTGGAACGAGACA
	Reverse	GCACGGAACAGAAGCTTTATTAGGA
CXCR2	Forward	CAATGAATGAATGAATGGCTAAG
	Reverse	AAAGTTTTCAAGGTTCGTCCGTGTT
CXCR3	Forward	CCCGCAACTGGTGCCGAGAAAG
	Reverse	AGGCGCAAGAGCAGCATCCACAT
CXCR4	Forward	ATCCCTGCCCTCCTGCTGACTATTC
	Reverse	GAGGGCCTTGCGCTTCTGGTG
CXCR5	Forward	TCCCCTCCTCACTCCCTTCCCATAA
	Reverse	CCTGCGGTTCCATCTGAGTGACATC
CXCR6	Forward	TTGTTTATAGCTTGCGCATTCTCAT
	Reverse	ATCCCCCTTGGTTTCAGCATTCTT
CX3CR	Forward	ATAGATTCCCCATTGCCTCCTC
	Reverse	GGTTTTTCTATTTCCCTTACTGG
β-actin	Forward	CCAACCGCGAGAAGATGA
	Reverse	CCAGAGGCGTACAGGGATAG

### Western blot

Whole-cell extracts were prepared by lysing cells in RIPA buffer [150 mM NaCl, 1% sodium deoxycholate, 0.1% sodium dodecyl sulfate (SDS), 50 mM Tris-HCl pH 7.4, 1 mM EDTA, 1 mM PMSF, and 1% Triton X-100] containing a cocktail of protease and phosphatase inhibitors. Equal amounts of each protein sample (30–50 μg) were separated by SDS-polyacrylamide gel electrophoresis (PAGE) and transferred to polyvinylidene fluoride membranes. The membranes were probed with primary antibodies against CXCR1, p-Akt, t-Akt, and GAPDH. The target proteins were detected using the Odyssey Infrared Imaging System (LI-COR Biosciences, Lincoln, NE, USA).

### Immunofluorescence staining

In brief, the cells were fixed with 4% paraformaldehyde, stained with rhodamine-linked CXCR1 primary antibody and Alexa 488-linked p-Akt primary antibody, and then counterstained with DAPI. Images of cells were captured using a confocal microscope (LSM510; Carl Zeiss).

### CXCR1 knockdown in Saos2-lung-M cells

Saos2-lung-M cells were transfected with lentiviral particles loaded with either small hairpin (sh)-RNA targeting CXCR1 (5′-TTCTAGGGATGCTGATGCT-3′) or a scrambled control (Thermo Fisher Scientific, Waltham, MA, USA) as described previously^25^.

### Animal models

Four-week-old nude mice (BALB/c, nu/nu; SIPPR-BK Laboratory Animal Co. Ltd, Shanghai, China) were housed under pathogen-free conditions at 26–28 °C with 50–65% humidity. All animal operations were approved by the Animal Ethics Committee of Shanghai Ninth People’s Hospital, Shanghai Jiao Tong University School of Medicine (A-2016-017). For intratibial injections, CXCR1-knockdown or scrambled control Saos2-lung-M cells were harvested, counted, and resuspended in PBS to a final concentration of 2 × 10^7^ cells/mL. In addition, RFP-labeled MSCs were harvested, counted, and resuspended in PBS to a final concentration of 4 × 10^7^ cells/mL. Trypan blue exclusion determined that >95% cells were viable prior to injection. The animals were anesthetized with 3.5% pentobarbital, and then 1 × 10^6^ OS cells and 2 × 10^6^ MSCs cells in 50 μL of PBS were injected into the proximal tibia using a 25-gauge needle. The tumor volumes were calculated using the formula: volume = 0.2618 × *L* × *W* × (*L* + *W*), where *W* and *L* represent the average width and length of the tumor, respectively^25^.

### Immunohistochemistry

The mice were sacrificed and tumor samples from each nude mouse were fixed in 4% paraformaldehyde, and then immunohistochemically stained for CXCR1 and p-Akt as described previously^41^.

### Statistical analysis

Statistical analyses were performed using SPSS software version 15.0 (SPSS Inc., Chicago, IL, USA). Data are presented as mean ± standard deviation. Comparisons between two groups were performed using Student’s *t*-test, while one-way analysis of variance was used for multiple comparisons. Analysis of survival and metastasis was performed using log-rank tests. Each sample was analyzed in triplicate and experiments were repeated thrice. *p* < 0.05 indicates a statistically significant difference.
